# Health Care Professionals’ Perspectives Before and After Use of eDialogue for Team-Based Digital Communication Across Settings: Qualitative Study

**DOI:** 10.2196/53391

**Published:** 2024-03-08

**Authors:** Lili Worre Høpfner Jensen, Ole Rahbek, Rikke Emilie Kildahl Lauritsen, Søren Kold, Birthe Dinesen

**Affiliations:** 1 Interdisciplinary Othopaedics Orthopaedic Surgery Department Aalborg University Hospital Aalborg Denmark; 2 Laboratory for Welfare Technologies-Digital Health and Rehabilitation ExerciseTech, Department of Health Science and Technology Aalborg University Aalborg Denmark

**Keywords:** CFIR, Consolidated Framework for Implementation Research, digital communication, hospital discharge, implementation science, interdisciplinary communication, orthopedic surgery, patient-provider communication, postoperative care, qualitative research, text messaging

## Abstract

**Background:**

Orthopedic surgical treatment is a transversal task that requires the active involvement of patients, relatives, and health care professionals (HCPs) across various settings. However, after hospital discharge, communication is challenged and undertaken primarily by phone. New digital communication solutions have the potential to create a space for seamless and patient-centered dialogue across discipline and sector boundaries. When evaluating new communication solutions, knowledge about HCPs’ needs and perspectives of use must be explored, as it is they who are responsible for implementing changes in practice.

**Objective:**

This study aimed to (1) investigate HCPs’ perceptions of current communication pathways (phase 1) and (2) explore their experiences of using a simple messenger-like solution (eDialogue) for team-based digital communication across settings (phase 2).

**Methods:**

We used a triangulation of qualitative data collection techniques, including document analysis, observations, focus groups, and individual interviews of HCPs before (n=28) and after (n=12) their use of eDialogue. Data collection and analysis were inspired by the Consolidated Framework for Implementation Research (CFIR) to specifically understand facilitators and barriers to implementation as perceived by HCPs.

**Results:**

HCPs perceive current communication pathways as insufficient for both patients and themselves. Phone calls are disruptive, and there is a lack of direct communication modalities when communication crosses sector boundaries. HCPs experienced the use of eDialogue as a quick and easy way for timely interdisciplinary interaction with patients and other HCPs across settings; however, concerns were raised about time consumption.

**Conclusions:**

eDialogue can provide needed support for interdisciplinary and cross-sectoral patient-centered communication. However, future studies of this solution should address its impact and the use of resources.

## Introduction

Treatment of patients undergoing orthopedic surgery is a cross-disciplinary task formed in partnership with the patient. Communication and collaboration between the patient and different professional groups across various settings are key to achieving quality in patient trajectories and clinical outcomes [1-3]. While hospitalization times are decreasing, an increasing part of the postoperative period takes place in the patient’s home and with support from municipal health care professionals (HCPs) [4,5]. However, they are largely dependent on contact with hospital staff when problems related to treatment and care arise.

In Denmark, the current means of communication between patients undergoing orthopedic surgery and HCPs across sectors is primarily by phone, but the synchronicity of this is inflexible and time-consuming. Moreover, HCPs across sectors communicate through different electronic systems, but without including patients in the dialogues. New communication strategies must aim to provide seamless communication paths that reach beyond the existing silos of the health care system and include patients as partners [6].

Digital patient platforms are being introduced in Denmark [7,8] as well as internationally [9,10]. Patients can receive digital patient education, see test results, and answer questionnaires used by clinicians to tailor treatment plans. In some cases, patients are given the opportunity to send texts in a secured chat to HCPs at the hospital before and after hospitalization in addition to phone calls. Internationally, secure messaging is reported as the most used feature on patient platforms [9]. Even though questions are not limited to nursing tasks, answering the messages is often delegated to nurses in outpatient clinics or wards at the hospital [7,8]. This leads to duplicate work for the nurses, who will act as intermediaries or gatekeepers for the questions that patients might have, in the same way as secretaries are gatekeepers for patient-initiated phone calls. Moreover, HCPs from the municipality are not involved in these digital encounters. Even though the surgeon at the hospital holds the primary responsibility for the orthopedic treatment [11], there are no direct communication modalities available between the patient, surgeon, and HCPs across sectors in the postoperative period. A team-based approach to the use of digital communication, involving the patient and all HCPs in their care team, may improve postdischarge communication and support patients more optimally after surgery and discharge. Our focus for this study was on communication pathways both involving patient-to-provider communication as well as provider-to-provider communication, as this is interwoven and interdependent in clinical practice.

In an exploratory qualitative study, we tested a simple messenger-like solution for team-based digital communication between patients and HCPs across sectors (eDialogue), and the perspectives of patients and their use of the solution have been reported in another study. However, when testing new communication pathways in health care, it is pivotal to explore the perspectives of all end users to identify their needs, motivations, and barriers to use at an early preimplementation stage [12]. Therefore, this study aimed to (1) investigate HCPs’ perceptions of current communication pathways with orthopedic surgery patients and collaborating HCPs across sectors, as well as their expectations for eDialogue (phase 1), and (2) explore their experiences of using eDialogue for team-based communication (phase 2).

## Methods

### Study Design

We used a triangulation of qualitative data collection techniques to understand contextual factors and what opportunities and challenges exist before (phase 1) and after (phase 2) the use of eDialogue. This included document analysis, observations [13], semistructured focus groups [14], and individual interviews [15]. Reporting this study followed the Consolidated Criteria for Reporting Qualitative Research (COREQ) checklist [16].

### Theoretical Framework

Conducting this study, we were inspired by the metatheoretical framework and terminology described by Damschroder et al [17]: the Consolidated Framework for Implementation Research (CFIR). The CFIR is widely used in health services research and specifically adapted to understand facilitators and barriers to implementation, even at an early preimplementation stage [17,18]. CFIR is centered around five key domains related to implementation, including (1) the intervention, (2) the inner setting, (3) the outer setting, (4) the individuals involved in the intervention, and (5) the processes conducted to implement the intervention [17]. To each domain belong underlying constructs, which describe factors that can either motivate or hinder implementation [17]. Selected CFIR domains and constructs guided our data collection by informing the interview guides and the observation protocol in combination with exploratory questions. In an inductive-deductive approach, CFIR domains and constructs were used to structure data analysis and the reporting of our findings, while still being open to emerging themes. By using CFIR, we aimed to promote structured knowledge building for future implementation strategies that may encourage the adoption of eDialogue in clinical practice.

### Participants and Setting

The study originated from the orthopedic surgery department at Aalborg University Hospital, which is a tertiary hospital in Denmark. The Danish health care system is mainly financed by general taxes and is therefore provided free of charge to individuals. It operates across 3 administrative and political levels, which are the state (national level), the regions (regional level), and the municipalities (local level). Hospital care is provided by the 5 regions of Denmark, and primary care and social services, such as rehabilitation outside hospitals, home nursing, and physiotherapy, are provided by the 98 municipalities of Denmark. Even though there is cofinancing and close collaboration between the regional and local levels, HCPs are employed in different organizations and use different electronic health records. There are defined care pathways for patients in need of treatment and care across settings that outline the tasks of the HCPs employed at the different levels, just as there is legislation that the HCPs must follow. However, major challenges exist in communication and collaboration across settings, especially related to patients in transitions of care from hospital to home.

#### Phase 1: Before eDialogue

In phase 1, orthopedic surgeons, secretaries, nurses, and physiotherapists from Aalborg University Hospital and home care nurses and physiotherapists from the Aalborg municipality were recruited for preintervention focus groups (n=6) to investigate their perceptions of current communication pathways and their expectations for eDialogue. Inclusion criteria were HCPs working with orthopedic patients from 2 different subspecialties that were recruited to test and explore eDialogue. These were patients undergoing either deformity correction surgery involving complex prolonged treatment with hospitalization or anterior cruciate ligament reconstruction performed as day surgery (ie, discharged on the day of surgery). HCPs were recruited from different units at the hospital, including the outpatient clinic, the ward, and the physiotherapy department, and from different districts of the Aalborg municipality. Exclusion criteria were HCPs who had sparse knowledge of orthopedic treatment and care; for example, personnel hired within the past year. We purposely strove to include HCPs from various vocational roles to achieve a detailed understanding of the clinical trajectory and interdisciplinary communication with patients undergoing orthopedic surgery. Inclusion persisted until data saturation was reached for the interviews, that is, no new themes occurred [15].

#### Intervention: eDialogue

**Figure 1:**
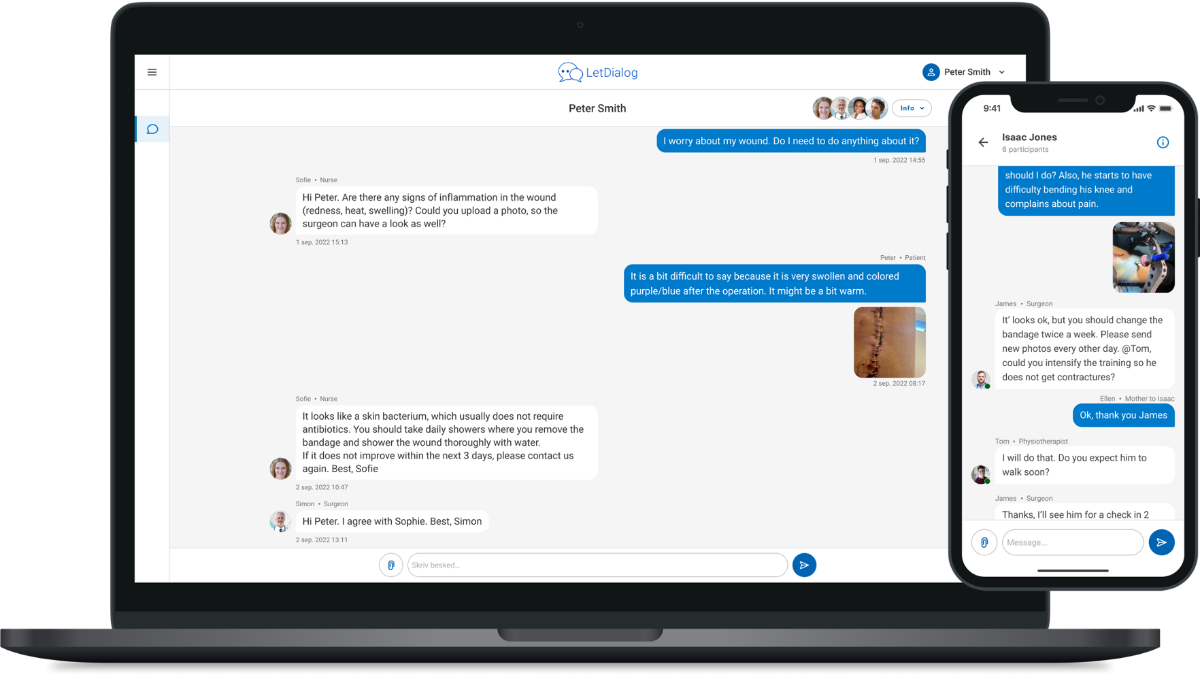
The figure shows screenshots of digital dialogues between patients and health care professionals (HCPs) across settings from the study. Access was either by app on a smartphone or by web, using a simple messenger-like user interface.

Team-based digital communication between patients and HCPs across settings was facilitated through a technical General Data Protection Regulation-compliant solution assessed by an app for a smartphone or through a website (Figure 1). The technical solution is already in use in some municipalities in Denmark in the field of social education [19], but has never been used to facilitate communication in health care or across sectors. The solution was chosen by the research team before the study based on the simple and intuitive interfaces and discussed with patients undergoing orthopedic surgery and HCPs in an initial workshop before this study.

Patients from 2 orthopedic surgery subspecialties were recruited consecutively for this study and offered to use eDialogue for 2 months after they had been discharged with their team of HCPs across settings.

Just as patients were helped to create an account using a digital signature (NemID), HCPs were guided to become users of eDialogue. Most HCPs accessed it through the website, but some preferred access through the app on their smartphones. Finger touch or face recognition could be used for login if access was through the app. During registration, all participants were given a short introduction to how to use eDialogue, including how to send texts and photos and get notifications of new posts. It was explained to HCPs that they were expected to provide answers to patients’ questions with a maximum response time of 24 hours on weekdays. In each individual case, patients decided which of the HCPs in their team of care they wanted to join the digital dialogue, and the HCPs were contacted and invited to join by the primary author (LWHJ). All communication was asynchronous, using text messages and photos; thus, no video calls could be made through the solution. Patients had access for 2 months after hospital discharge. Upon request and agreement with their team of HCPs, access could be extended beyond the study period. The digital dialogues were stored in a secure cloud-based solution [19], and a data processor agreement was made before the study.

#### Phase 2: During and After Use of eDialogue

HCPs were recruited for interviews after their use of eDialogue with patients and other HCPs across disciplines and sectors. The inclusion criteria were involvement in eDialogue with ≥3 patients. There were no exclusion criteria.

### Data Collection

**Figure 2:**
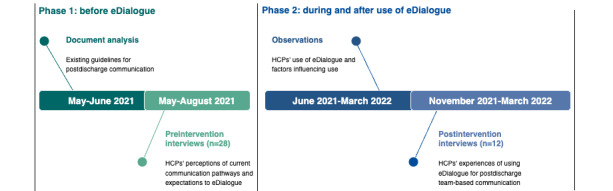
The figure illustrates the timeline and data collection for phases 1 and 2 of this study. In phase 1, document analysis and preintervention interviews with health care professionals (HCPs) were performed. In phase 2, observations were conducted continuously as eDialogue was used, and, ultimately, postintervention interviews were performed.

Data collection was structured according to the two phases to achieve thorough insight into HCPs’ perceptions of current communication pathways and their expectations of eDialogue before use (phase 1), and to explore their experiences with access to eDialogue (phase 2). Figure 2 illustrates the triangulation of data collection techniques across the 2 phases of this study.

#### Phase 1: Document Analysis and Preintervention Interviews

An initial document analysis of existing guidelines for communication between patients undergoing orthopedic surgery and HCPs across sectors was carried out with the aim of gaining insight into the current context for communication. First, we identified relevant practical documents by searching different Danish web pages related to the political and regulatory guidelines on transitions of care from hospital to home and strategies for using information technologies in health care, for example, the Ministry of Health, the Local Government of Denmark, and the Danish Society for Patient Safety. We also searched the local web page of Aalborg University Hospital for clinical practice guidelines describing the procedures that HCPs must follow when patients or municipal providers contact them regarding discharged patients. Second, we applied a snowball strategy, using references from the initial search. We did not formally analyze the documents, but we used knowledge of the context to understand the framework under which HCPs must work and to qualify the interview guide.

This was followed by focus groups with HCPs across the hospital and municipality (n=28). The aim of the focus groups (n=6) was to explore HCPs’ perceptions of current communication pathways and their expectations of eDialogue before use.

The interview guide was inspired by the CFIR Interview Guide Tool [20], including exploratory questions to provide space for emerging reflections. The interview guide was tested on 2 HCPs from the hospital and discussed among the authors until agreement was reached. Minor additions were made before the first focus group.

All preintervention interviews were conducted as semistructured focus groups, dividing HCPs according to their vocational roles and setting (hospital or municipality). HCPs interviewed were surgeons at the hospital (n=5), secretaries from the hospital (n=3), nurses from the hospital ward (n=5), nurses from the outpatient clinic (n=3), home care nurses from the municipality (n=3), physiotherapists from the hospital (n=5), and physiotherapists from the municipality (n=4). Using preexisting groups as focus groups was based on the assumption that it would make participants discuss and compare their reflections in depth in the same context and without an underlying power structure that could occur if professions were mixed [14]. At the beginning of each interview, background variables such as gender, vocational role, and years of experience with patients undergoing orthopedic surgery were collected.

All interviews with HCPs from the hospital were conducted face-to-face by the first author (LWHJ). For the first 2 focus groups, a project nurse was present to register observations during the interviews and to take notes to qualify and supplement the interview. Focus groups with HCPs from the municipality were performed remotely by video, as data collection occurred during the coronavirus outbreak and most HCPs outside of the hospital were not physically located in the same place. The interviewer summarized key points during and at the end of each focus group to facilitate further reflection and to make sure her interpretation corresponded with what the HCPs had said [15]. Field notes were made at the end of each focus group so as to remember details of the context, group interaction, and nonverbal communication [15]. The focus groups lasted an average of 1 hour (between 45 and 90 minutes).

#### Phase 2: Observations and Postintervention Interviews

In total, eDialogue was used with 31 patients and with the involvement of 24 different HCPs. When the last patient had had access to eDialogue with their team of HCPs for 2 months, a convenience sample of participating HCPs across the hospital and municipality were interviewed (n=12), including surgeons from the hospital (n=5), physiotherapists from the hospital (n=2), and from the municipality (n=5). We performed 7 individual interviews with physiotherapists across hospitals and municipalities and 1 focus group with 5 surgeons. The aim of the interviews was to explore their experiences with eDialogue. All interviews were conducted by LWHJ, audio recorded, and followed a predefined semistructured interview guide inspired by the CFIR Interview Guide Tool [20] and additional exploratory questions. Interviews with HCPs from the hospital were performed face-to-face, and interviews with HCPs from the municipality were conducted remotely based on the participants’ wishes.

During the study period, we observed the use of eDialogue by HCPs and documented this in Word (Microsoft Corporation) files. The aim was to observe issues related to HCPs’ use of eDialogue that were reported to the project group or observed in dialogues (an administrator from the project group was present in all dialogues to observe if eDialogue was used in acute situations). HCPs were encouraged to contact the first author if they experienced any problems with eDialogue or had concerns or questions during use, and these were documented as well. Data collected through observations were used to qualify the follow-up interviews in phase 2 and were also imported to NVivo (QSR International) for analysis in conjunction with interview data.

### Data Analysis

Data were analyzed for phase 1 and then phase 2, respectively. Interviews were audio recorded using a digital voice recorder (DM-450; Olympus) and transcribed verbatim immediately afterward. Word files with the transcriptions were imported to NVivo for data analysis (NVivo 12, version 20.6.2) [21]. Inspired by Brinkmann and Kvale [15], using an inductive-deductive approach, we performed thematic analysis focusing on meaning (Textbox 1).

**Steps of the thematic analysis using an inductive-deductive approach.**

**Meaning coding**

- Full transcripts were read several times by both LWHJ and REKL.
- To define the initial coding template and to achieve intersubjectivity, the first 4 interviews of each phase were coded by LWHJ and REKL individually before meeting to compare and discuss codes until mutual agreement was achieved. When the coding template was defined, LWHJ applied the same codes to the entire data set. The approach to this step was inductive, thus reflective of the issues raised in the data set.

**Meaning condensation**

- Theme development was undertaken with a more deductive approach, where domains and constructs from the Consolidated Framework for Implementation Research (CFIR) were used to organize the codes and inform theme development to specifically focus on facilitators and barriers to eDialogue. However, in developing themes, we were open to emerging themes that did not fit the CFIR domains and constructs. Codes and themes were reread and revised by LWHJ in collaboration with REKL and BD in several iterations.

**Meaning interpretation**

- Definitions and narrative descriptions of themes were made. Data extracts were selected to be presented in the manuscript.
- The final analysis and description of the findings were written.

Data analysis was conducted separately for phase 1 and phase 2 following the 3 steps of meaning coding, meaning condensation, and meaning interpretation. In phase 2, we added notes from observations to the data set to achieve an in-depth understanding of the context in which HCPs had used eDialogue and any problems occurring during use.

### Ethical Considerations

The Ethics Committee of Northern Jutland was contacted before the start of the study. They decided by email on March 18, 2021, that the study did not require approval (journal number 2021-000438), as the intervention would not have consequences for diagnostics or treatment. We registered the study at the Regional Committee on Health Research (ID 2021-057). The study followed the Helsinki Declaration, and all participants received both oral and written information as well as thorough guidance in the use of eDialogue. To take into account patients’ possible use of eDialogue in emergency situations, an administrator was present in all digital dialogues.

## Results

### Participant Characteristics

In phase 1, a total of 28 HCPs were recruited across vocational roles and hospital and municipal settings (Table 1). All surgeons, nurses, physiotherapists, and secretaries from the clinical orthopedic surgery subspecialties at the hospital, from which the patients were recruited (deformity correction or anterior cruciate ligament injury), were invited to participate in interviews. However, 2 surgeons, 1 nurse from the outpatient clinic, 1 nurse from the municipality, and 3 secretaries were not able to. Nurses from the ward were purposefully selected based on years of experience and a pragmatic approach to who would be able to participate in interviews during their work hours. On average, HCPs had 11 (range 1-30) years of experience with patients undergoing orthopedic surgery.

**Table 1:** Vocational roles of health care professionals who were interviewed in phases 1 and 2.

Vocational role	Phase 1 (N=28), n	Involved in eDialogue (n=24), n	Phase 2 (n=12), n
Orthopedic surgeon, hospital	5	7	5
Nurse, outpatient clinic, hospital	3	1	N/A^a^
Nurse, ward, hospital	5	N/A	N/A
Physiotherapist, hospital	5	5	2
Secretary, hospital	3	N/A	N/A
Physiotherapist, municipality	4	11	5
Nurse, municipality	3	N/A	N/A

^a^N/A: not applicable.

In phase 2, a total of 12 HCPs were included for interviews, of whom 8 had also participated in focus groups in phase 1. The HCPs recruited at this stage were a sample of those who had experiences with communication in eDialogue (Table 1). Of whom, 8 HCPs interviewed for phase 2 had also participated in focus groups in phase 1. In total, 24 HCPs across the hospital and municipality were involved in eDialogue. However, we prioritized including those who had been set up to communicate in eDialogue with ≥3 patients. One nurse from the outpatient clinic had been involved in 3 dialogues but was not able to participate due to being absent at the time of the interviews. No nurses from the ward or the municipality were users of eDialogue and thus were not interviewed in phase 2. Secretaries were not interviewed in phase 2, as we decided not to include them in eDialogue at this point.

On average, there were 3.3 (range 2-4) HCPs per patient in the dialogues. All patients were at least connected with the orthopedic surgeon, and 25 of 31 patients had their municipal or hospital-based physiotherapist involved as well.

### Themes and Subthemes Identified in Phases 1 and 2

In Table 2, the findings of the analysis of phases 1 and 2 are presented together in main themes organized by the CFIR domains and constructs and additional subthemes. This is to display the before-and-after perspectives of HCPs. Following the table, we elaborate on subthemes in narrative text according to phases 1 and 2 and by using selected quotes from interviews.

The main themes are organized by CFIR domains and constructs, and subthemes elaborate on these for phases 1 and 2, respectively. Emerging themes occurred in both phases that did not match any of the CFIR constructs, and they are therefore described under additional emerging themes.

**Table 2:** Themes and subthemes from phases 1 and 2 organized by the Consolidated Framework for Implementation Research (CFIR) and additional emerging themes.

Main themes: CFIR domains and constructs		Subthemes	
		Phase 1: before eDialogue	Phase 2: after eDialogue
**Intervention characteristics**			
	Relative advantage	Contradictory expectations for using eDialogue versus phone call A lifeline and reassurance for both patients and HCPs^a^ Hidden work can become visible	Quick and easy to interact in eDialogue Photos in eDialogue improve the quality of communication
	Adaptability	N/A^b^	Development of individual strategies and workflows for the use of eDialogue
**Outer setting**			
	Needs and resources	Patients are messengers of information between HCPs	Timely and effective interdisciplinary communication with patients across settings
**Inner setting**			
	Tension for change	Feeling like an insufficient intermediary Phone calls are disruptive, yet necessary	N/A
	Relative priority	Experiences of technology fatigue	N/A
	Compatibility	N/A	Divergent perceptions of how well eDialogue meets needs
	Available resources	N/A	Concerns about resource consumption Need for clarification regarding financial incentives
**Characteristics of individuals**			
	Self-efficacy	N/A	To express oneself in writing
**Additional emerging themes**			
	Previous experiences with digital communication	Email and SMS text messaging are already used with patients and for interdisciplinary communication; however, standardization is lacking	N/A
	Reflection and learning	N/A	A new space for studying patients’ needs

^a^HCP: health care professional.

^b^N/A: not applicable.

#### Phase 1: Current Communication Pathways and Expectations for eDialogue

##### Intervention Characteristics

Even though the majority of HCPs expected eDialogue to provide optimized interdisciplinary communication, prevent conflicting recommendations to patients, and provide easier access for patients, there were contradictory expectations for the use of eDialogue. On one hand, HCPs had concerns about whether answering messages would require more of their time and go beyond working hours, but on the other hand, they thought it would be easier to answer in eDialogue than by phone. Concerns also centered around whether using text as a communication medium would be adequate for all patients and if misunderstandings would occur due to wrong interpretations. HCPs were especially worried about whether they would pick up on complications to the same extent as they do by phone.

I can't hear the patient's voice answering back, and if they have understood my answers (…) however, it depends on the complexity of their questions, whether it's just how many repetitions was it, or something that could be more serious.

Physiotherapist, municipality, preintervention interview

Being able to send photos in eDialogue was expected to be an important feature that might offset the challenges of using text for communication. Even though some HCPs had reservations and conflicting opinions about eDialogue before its use, they all agreed that it would be a reassurance and a lifeline for both patients and HCPs across settings. Additionally, a physiotherapist from the hospital reflected on how eDialogue might bring “hidden tasks” to light.

What I thought at first would be negative like, oh then we have to do that too, will probably actually be reversed, so that the hidden tasks, we solve by calling and writing notes and emails and things like that, becomes more visible and can be accounted for during work hours.

Physiotherapist, hospital, preintervention interview

##### Outer Setting

The analysis revealed that HCPs experience current communication pathways in the postoperative period to be challenging, both by phone and existing electronic systems. This leads to workarounds, such as HCPs giving patients oral instructions or written notes to bring to other HCPs to ensure timely information. However, using patients as messengers of information between HCPs is perceived as insufficient yet necessary in current communication pathways. A physiotherapist from the municipality described how current systems do not support the patient’s trajectory across sectors.

There are watertight shutters between the communication systems, i.e., what they write in the medical record at the hospital and what I write here. The surgeon at the hospital can't see that, and (…) I can't see his note.

Physiotherapist, municipality, preintervention interview

##### Inner Setting

Across professional roles and settings, HCPs expressed a need for change to enable easier sharing of knowledge and communication. This was especially the case for complex and long-term orthopedic surgical treatments where multiple HCPs are involved. Knowing each other across settings, for example, by being former colleagues, was a mediating factor for communication between HCPs. However, it was not perceived as sustainable.

Getting in contact with each other and patients by phone is considered time consuming due to the synchronicity of phone calls. A nurse from the inpatient department described how phone calls would sometimes be left until the next day if questions required the involvement of another HCP. This left the nurses feeling like inadequate intermediaries and could be a risk to patient safety. Similar experiences were described by physiotherapists, who often found themselves being asked about issues outside of their competencies; for example, questions about wounds and medication.

HCPs from the hospital described how phone calls are disruptive to their work processes, even though they understood the need for them. In addition to inquiries from patients, they receive phone calls from a wide range of HCPs in hospitals, municipalities, and private settings. Although secretaries act as gatekeepers, nurses from the outpatient clinic, and in the inpatient department in particular, handle many phone calls daily.

It's constant, isn't it? (...) it takes my attention away from the dialogue, the communication and the relationship that I’m in the middle of. Then you’re like, oh sorry, this phone call is actually more important than you are (the patient they are with).

Nurse, outpatient clinic, hospital, preintervention interview

Addressing eDialogue as a novel communication solution to support team-based communication between patients and HCPs across settings, most HCPs were positive about the change it might bring. However, they expressed some degree of technology fatigue that made them skeptical of yet another system without integration into existing systems.

##### Emerging Theme

HCPs described previous experiences with using digital communication with patients, usually by email or SMS text messaging. Most often, it is used as a way to provide psychological reassurance to patients or to solve specific complex problems, where the HCPs have specialist knowledge. Even FaceTime was described as being used once with a patient to inspect a wound from a distance. However, the disadvantages of the current nonsystematic use of digital communication with patients were reflected. Concerns were raised regarding using a private phone number and the risk of introducing data security breaches. Also, giving some patients the opportunity for direct digital contact and others not was perceived as problematic. Thus, if used inconsistently, it may lead to inequality in patients’ access to health care.

Furthermore, HCPs described how they use email or SMS text messaging to communicate with each other, for example, to share thoughts on treatments or rehabilitation. They do this as a workaround to traditional communication pathways or because it is perceived as less disturbing to each other. Thus, the use of digital text-based communication is not uncommon for HCPs in this study. However, it is not standardized or even articulated among colleagues or management.

#### Phase 2: HCPs’ Perspectives of eDialogue After Use

##### Intervention Characteristics

All HCPs agreed that the technical solution for eDialogue was very intuitive and did not need a thorough introduction, as opposed to other solutions with more features. Most HCPs articulated that questions were quick and easy to handle during work hours. Especially the asynchrony of the contact and the use of photos improved the quality of communication and their experiences of eDialogue for patient communication.

The big advantage of this, is that they can send a photo (…). If it wasn't a possibility, I think there would be a lot of writing about something that we couldn't really clarify, and then we would still have to call them in (for an extra check). Being able to send a photo, that’s really crucial for this to work.

Surgeon, hospital, postintervention interview

The analysis demonstrated that HCPs developed individual strategies for answering questions in eDialogue. Notifications were automatically sent to participants when there were new messages in the system, but there were no integrated reminders to follow up if the messages were not read within 24 hours, and this led to the development of individual workflows.

(The notification) on email, when there is a new message, I will not delete it until I have answered. That way, it helps me keep track.

Surgeon, hospital, postintervention interview

eDialogue was mainly used by patients as a place to ask postdischarge questions to HCPs. In general, most questions from patients were answered by surgeons and physiotherapists from the hospital. Municipal physiotherapists described being hesitant to involve themselves actively in answering, as they experienced hospital staff being quick to answer the patients. However, they emphasized that they used the information given to the patient by hospital staff in their subsequent contact with patients. This “indirect” use was perceived as valuable to them.

It has been very rewarding to just follow the dialogue, even though I was not active in it. The fact that the patient can just send a photo and ask ‘what does this look like?’, then he is immediately calmed down. It's rather smart, and also that I know of it right away.

Physiotherapist, municipality, postintervention interview

##### Outer Setting

HCPs stressed that the team-based approach made it easier to share timely information with the patient and other HCPs, and thereby it created more effective communication pathways. Physiotherapists highlighted how their previous perceptions of being an insufficient intermediary between the patient and other HCPs were changed when communication could take place directly in eDialogue.

It was actually really nice that he (the patient) just took it directly with the surgeon. Because I can have doubts (…) and you don’t want to burden the surgeon by calling.

Physiotherapist, municipality, postintervention interviews

##### Inner Setting

Even though HCPs acknowledged the impact that eDialogue had for patients, there were discrepancies in their perception of how it was used in this study, and it affected their acceptance of the solution. For example, some HCPs thought that the team-based approach was not necessary for all patients involved or that they lacked a secretary for administrative tasks. As such, they highlighted that some questions might be better answered in other ways, for example, by providing better patient education or by including other HCPs in the dialogue.

I think it is difficult to say that the patients' questions are not relevant because they must be since they ask them, but who should answer them, and how quickly should they have an answer, can be discussed.

Physiotherapist, hospital, postintervention interview

However, when using eDialogue with patients for complex orthopedic treatments, HCPs expressed that the team-based approach was very valuable to the patients and their workflows.

I think it was good. They (patients) feel that there is a team around them, and I get the feeling that I’m not the only one being responsible. Also, I don't have to spend time calling the physiotherapist to say ‘Hey, can't you just look at this?’ when he’s already in the dialogue.

Surgeon, hospital, postintervention interview

HCPs strongly experienced that access to eDialogue provided reassurance for patients. However, in consideration of the sparse health care resources, it was a general opinion that eDialogue should only be offered to patients for complex treatments. This provoked an ethical discussion of how HCPs could distinguish between who should be offered the solution and who should not. HCPs highlighted that an assessment of effects should be addressed, both in terms of resource consumption and patient outcomes.

One of my concerns with systems like this is that if we have to use it with all patients (…), then I think it could become a burden. And also, I think it will be difficult to say, well, it's only for some patients, because why them?

Surgeon, hospital, postintervention interview

HCPs agreed that clarification is needed regarding financial incentives before implementing eDialogue. Along with concerns about resources to answer the questions, this was a perceived barrier to use.

I think the barriers are time and finances (...) there is, of course, someone who looks at what I produce. And I think it should be some kind of service that should be visible (to others), if we have to evaluate a photo or send back a response (through eDialogue).

Physiotherapist, municipality, postintervention interview

##### Characteristics of Individuals

In all interviews, HCPs had concerns about whether they expressed themselves clearly enough in writing and how their “tone of voice” would be perceived by patients when formulated in texts. In reflection, they emphasized that the same concerns could arise when talking to patients on the phone.

Regardless of whether it's something you say to them or something you text them, it's just as important that you use words they can understand, and I actually often think it's a little easier when you text because you have time to think about it.

Surgeon, hospital, postintervention interview

There were clear differences in how HCPs expressed themselves in the texts, and this was discussed in one of the focus groups, where a surgeon had been involved in another surgeon’s dialogue due to vacation.

I think he (the other surgeon) is very kind in his feedback. I actually noticed that, you (addressed to the other surgeon) have formulated yourself in such a very friendly way, in contrast to what I did to start with. I made it very short, like I might normally answer a text message with a friend (…). I had to remind myself that they don't know me (…) it might be important to pay attention to that.

Surgeon, hospital, postintervention interview

##### Emerging Theme

Both surgeons and physiotherapists described that using eDialogue created interdisciplinary reflection and learning about patients’ needs after discharge, and that frequently asked questions could be used to improve future patient education.

It gives feedback in relation to the material we use and the way we inform patients now. It might actually be very nice for all of us to know this.

Physiotherapist, hospital, postintervention interview

Ultimately, HCPs pointed out that they could learn from each other by reading each other’s answers to patients.

## Discussion

### Principal Findings and Comparison With Previous Work

This study first investigated HCPs’ perceptions of current communication pathways with patients and other HCPs involved in the patient’s trajectory after orthopedic surgery and discharge, along with their expectations for eDialogue before its use (phase 1). Following initial document analysis, we included a wide range of HCPs across vocational roles and settings in focus groups to obtain an in-depth understanding of their needs and attitudes toward eDialogue. These perspectives are important to capture, as individual and contextual factors as well as initial perceptions of eDialogue may motivate or hinder use [17]. The findings of phase 1 showed that, on the one hand, HCPs perceived a significant tension for change. Current communication pathways are perceived as insufficient, phone calls are disruptive, and patients unfortunately become messengers of information between HCPs across settings. On the other hand, HCPs expressed conflicting attitudes toward eDialogue in advance of its use. Positive or negative attitudes were not limited to certain vocational roles but were expressed in all groups and also as an internal dilemma inherent to the individual. However, there were clear expectations for eDialogue to support patients in the postoperative period and consensus that it may provide optimized interdisciplinary and cross-sectoral communication. At the same time, HCPs experienced some degree of technology fatigue and significant worry that eDialogue would be time-consuming for them to handle.

Second, we explored HCPs’ experiences of using eDialogue for team-based digital communication through observations and postintervention interviews (phase 2). Knowing that, even with highly developed plans for execution, undiscovered factors can undermine implementation efforts in the real world [17,18], we searched to identify facilitators and barriers to implementation from the perspectives of key users at an early stage. Findings from phase 2 showed that HCPs experienced eDialogue as a quick and easy way to interact with patients and other HCPs and that eDialogue could support timely and effective interdisciplinary communication across settings. As such, the positive perceptions of the importance of eDialogue described in the preintervention interviews were maintained. Similarly, the use of photos was expected to be important in preintervention interviews, and in postintervention interviews, photos were even suggested as being a significant quality-enhancing element compared to traditional phone calls. Similar findings have been described in other studies of digital communication in health care [22-24].

In interviews in phase 1, HCPs described that they had concerns about communicating with patients in texts because they feared overlooking an important complication or that the patient would misunderstand their written responses. In phase 2, HCPs still expressed concerns about whether they expressed themselves clearly enough. However, they pointed out that the same risks can be present in phone consultations. This perspective is supported by a recent study of telephone consultations in Denmark. Jensen et al [25] found that communication in consultations concerning back pain preceding out-of-hospital cardiac arrest was influenced by the communicative preconditions of the call-taker, thereby addressing the fact that a meaning-constitution is undertaken in the interaction between the patient and the call-taker, not always reflecting the actual problem. To learn from this, HCPs involved with patients through eDialogue and other digital communication solutions must be aware that communicative interaction is always an interpretative task for the receiver of a message. Even though the HCPs’ concern might decrease as they gain more experience communicating in writing, their self-efficacy should be supported by formulating clear recommendations, training, and supervision.

Across the interviews of phases 1 and 2, HCPs expressed concerns regarding resource consumption; this was particularly evident among hospital staff. While acknowledging patients’ need for easier access to communication with HCPs after discharge, HCPs questioned if the team-based approach was necessary for patients undergoing less complex orthopedic treatments. Nevertheless, there was consensus that eDialogue can support patients in complex and long-term treatments and that a needs assessment to learn who will benefit the most from eDialogue should be made before its implementation so as to best match resources with actual needs. Other studies investigating the use of team-based digital communication have primarily focused on patients with cancer or chronic diseases [26-29]. Patients undergoing orthopedic surgery for complex and long-term treatment suffer similar challenges in health care communication [30], and therefore it is also relevant to develop and test solutions for this group. By using eDialogue for a smaller patient group, the workload caused by the implementation of the solution will decrease.

eDialogue was a solution where both patients and HCPs across settings could communicate freely in the postdischarge period. However, the primary communication in eDialogue was between the patient and HCPs at the hospital. Municipal physiotherapists used eDialogue more indirectly as a way to keep up to date with the patients’ progress. As such, findings revealed how physiotherapists in the municipality and patients together would formulate questions to send to the hospital staff. Taking into account this shared use of eDialogue, usage data defining the proportion of messages sent between patients and HCPs and between HCPs across settings would not be representative of their actual use. Moreover, HCPs adapted eDialogue to their contexts and developed individual strategies for providing timely answers. Some strategies were developed because the technical solution lacked better adaptation to the context, for example, an improved notification system, whereas other strategies were based on individual preferences in handling digital communication. All cases emphasize the importance of uncovering the HCPs’ context and needs and ensuring that new technology supports them in their work processes so that inappropriate use of new solutions does not end up adding new workarounds and thus hindering the optimal outcome of the technology.

### Limitations

This study was inspired by the CFIR to guide data collection and analysis [17,20]. The systematic identification and mapping of what was perceived as important to HCPs to the CFIR domains and constructs was helpful in providing an overview of the multifaceted and conflicting attitudes and experiences of eDialogue. However, we did not apply the CFIR as exhaustively as recommended [17,18], and we may thereby have missed important aspects that could have emerged. Using an inductive-deductive approach in data analysis, however, allowed us to still be explorative, which suited the early phase of the intervention described in this study.

In phase 1, we included a wide range of HCPs involved in the patients’ trajectory and communicative circles after surgery and discharge to shed light on their perspectives on current communication pathways. Including HCPs from different settings was a strength to this study, however, the small subgroups of HCPs from the same setting may jeopardize data saturation [15]. However, the theme of the interviews, exclusively focusing on communication, is narrow and may thereby outweigh this issue. For preintervention interviews, data saturation was reached; however, it can be discussed whether data saturation was reached fully for the interviews in phase 2. Observations of HCPs’ use of eDialogue, including technical or collaborative issues that were encountered during use, accounted for this and were included in the data analysis for phase 2.

Furthermore, we could have included management and decision makers in the focus group to gain a deeper understanding of the political and managerial context of the use of eDialogue across sectors. However, this was not attempted in this study as we wished to focus on the end users’ perspectives.

Our findings derive from a single hospital and a municipal region in Denmark. Therefore, they may not reflect the experiences of HCPs from other parts of the country, where different digital communication solutions have been implemented. Only 1 nurse participated in communication in eDialogue, and thus the experiences of this group of HCPs are not reflected in our findings. Unfortunately, at the start of this study, the coronavirus outbreak was at its peak, and many nurses from the hospital were reassigned to newly opened COVID-19 departments. At the same time, there was a trade union strike among nurses in Denmark, which resulted in the cessation of work for a period of time for many nurses from the municipality. These circumstances put greater work pressure on the nurses, and we continued the study without their active involvement in the dialogues.

Last but not least, some of the research team members behind this study are clinicians and were involved in the decision to test eDialogue. We have tried to overcome this issue by including research team members with little knowledge of the patients and processes in orthopedic surgery. Thus, 2 independent researchers coded and condensed data (LWHJ and REKL) in close discussion with BD, where REKL did not have preliminary knowledge of the context.

### Conclusions

HCPs describe current communication pathways as complicated. Phone calls are disruptive to work processes, and the lack of direct communication modalities between patients and HCPs across settings in the postoperative period makes patients become messengers of information between HCPs. To overcome these challenges, HCPs use off-the-shelf digital communication solutions as a workaround; however, use is neither standardized nor data secure. HCPs were open to using eDialogue, although they had reservations, which were partly confirmed and unconfirmed in their subsequent use of eDialogue. Especially, concerns regarding resource consumption were highlighted, and HCPs suggested the solution is particularly valuable in complex and prolonged treatments. The use of eDialogue offers a potentially valuable strategy for future integration of communication across health care settings, breaking down existing silos and taking into account the whole care team and the patient. This study provides knowledge for future strategies for implementing such solutions in orthopedic surgery and other clinical domains.

## Abbreviations

CFIR: Consolidated Framework for Implementation Research
COREQ: Consolidated Criteria for Reporting Qualitative Research
HCP: health care professional
